# Expression of Protein-Coding Gene Orthologs in Zebrafish and Mouse Inner Ear Non-sensory Supporting Cells

**DOI:** 10.3389/fnins.2019.01117

**Published:** 2019-10-18

**Authors:** Kimberlee P. Giffen, Huizhan Liu, Kenneth L. Kramer, David Z. He

**Affiliations:** Department of Biomedical Sciences, Creighton University School of Medicine, Omaha, NE, United States

**Keywords:** ortholog analysis, protein-coding genes, inner ear, supporting cells, hair cell regeneration, zebrafish, mouse

## Abstract

Non-mammalian vertebrates, including zebrafish, retain the ability to regenerate hair cells (HCs) due to unknown molecular mechanisms that regulate proliferation and conversion of non-sensory supporting cells (nsSCs) to HCs. This regenerative capacity is not conserved in mammals. Identification of uniquely expressed orthologous genes in zebrafish nsSCs may reveal gene candidates involved in the proliferation and transdifferentiation of zebrafish nsSCs to HCs in the inner ear. A list of orthologous protein-coding genes was generated based on an Ensembl Biomart comparison of the zebrafish and mouse genomes. Our previously published RNA-seq-based transcriptome datasets of isolated inner ear zebrafish nsSCs and HCs, and mouse non-sensory supporting pillar and Deiters’ cells, and HCs, were merged to analyze gene expression patterns between the two species. Out of 17,498 total orthologs, 11,752 were expressed in zebrafish nsSCs and over 10,000 orthologs were expressed in mouse pillar and Deiters’ cells. Differentially expressed genes common among the zebrafish nsSCs and mouse pillar and Deiters’ cells, compared to species-specific HCs, included 306 downregulated and 314 upregulated genes; however, over 1,500 genes were uniquely upregulated in zebrafish nsSCs. Functional analysis of genes uniquely expressed in nsSCs identified several transcription factors associated with cell fate determination, cell differentiation and nervous system development, indicating inherent molecular properties of nsSCs that promote self-renewal and transdifferentiation into new HCs. Our study provides a means of characterizing these orthologous genes, involved in proliferation and transdifferentiation of nsSCs to HCs in zebrafish, which may lead to identification of potential targets for HC regeneration in mammals.

## Introduction

The auditory organ in the vertebrate inner ear contains sensory HCs and non-sensory supporting cells. HCs are sensory receptor cells with a characteristic stereocilia bundle on the apical surface that converts mechanical stimuli into a receptor potential. Supporting cell populations are critical for maintaining ion gradients, facilitating removal of glutamate from synapses, and promoting survival of HCs (Slepecky, 1996; Raphael and Altschuler, 2003; Monzack and Cunningham, 2013). Supporting cells in the mammalian organ of Corti are terminally differentiated, highly organized, and morphologically distinct cells, while supporting cells in the inner ear of non-mammalian vertebrates are less so (Groves et al., 2013).

Examination of vertebrate hair cells (HCs) has revealed a number of genes associated with their differentiation, development, and function as mechanoreceptors in the detection of sound (Nicolson, 2005; Nayak et al., 2007; Fritzsch et al., 2011). Conservation of function among gene orthologs within cells of the inner ear is evidenced by the known deafness genes expressed in mammalian and zebrafish HCs (see Whitfield et al., 1996, 2002; Nicolson, 2005; Brignull et al., 2009). Additionally, HCs in both mammals and non-mammals are highly vulnerable to damage from exposure to noise and ototoxic drugs. Despite apparent homologies, non-mammalian vertebrates, including zebrafish and chicken, retain the ability to spontaneously regenerate HCs through proliferation and conversion of nsSCs to HCs (Brignull et al., 2009; Abbas and Whitfield, 2010; Millimaki et al., 2010; Monzack and Cunningham, 2013). In contrast, supporting cells in the adult mammalian cochlea no longer retain that capability (Géléoc and Holt, 2014). The absence of this innate regenerative mechanism in adult mammals leads to permanent hearing loss following hair cell death.

The molecular mechanisms allowing non-mammalian supporting cells to proliferate and transdifferentiate into HCs are poorly understood. Comparing the gene expression profiles of non-mammalian and mammalian supporting cells will provide essential information to understand the molecular mechanisms of HC regeneration. Transcriptomic analysis reveals unique cellular phenotypes and intrinsic properties of the cells in the inner ear, including expressed transcription factors and other molecular signals that regulate gene expression and cellular function. The goal of the present study was to identify protein-coding gene orthologs, including transcription factors, that are differentially or uniquely expressed in zebrafish and mouse inner ear supporting cells. Recent RNA-seq studies characterized the transcriptomes of adult zebrafish inner ear nsSCs (Barta et al., 2018), as well as PCs and DCs of the organ of Corti (Liu et al., 2018). Of the five types of supporting cells in the mammalian cochlea, PCs and DCs are likely targets for HC regeneration. This is due to their proximity to hair cells and ability to transdifferentiate to HC-like cells when Atoh1 is overexpressed or when Notch signaling is inhibited in the neonate cochlea (Izumikawa et al., 2005; Shi et al., 2012, 2013; Mizutari et al., 2013; Cox et al., 2014; Li et al., 2018). Our comparison of the protein-coding gene ortholog expression profiles of fully differentiated PC and DC populations to zebrafish nsSCs identified the similarities underlying non-mammalian and mammalian inner ear supporting cells, while also spotlighting some of the essential differences that underlie the lack of inherent supporting cell to HC conversion in mammals.

Our comparative analysis of the zebrafish and mouse genomes identified 17,498 protein-coding gene orthologs in zebrafish corresponding to 13,557 orthologs in mice. The commonly upregulated protein-coding gene orthologs in zebrafish nsSCs, compared to mouse PCs and DCs, indicated many conserved functions among these cell populations. Additionally, the analysis revealed that PCs and DCs shared over 300 upregulated gene orthologs with zebrafish nsSCs involved in biological processes of cell proliferation, epithelium differentiation, embryo development, and neurogenesis. Over 1,500 genes were identified as uniquely upregulated in zebrafish nsSCs, many of which may contribute to the stem cell-like properties of this cell population. Our study yielded two significant findings. First, we found that numerous genes expressed only in the zebrafish nsSCs are known to play a role in differentiation and development of HCs and supporting cells in vertebrates including: *foxi1*, *gli1*, *neurog1*, *six3a*, *zic1*, and *zic3*. Second, several genes uniquely or highly expressed in zebrafish nsSCs are classified as stem cell markers or transcription factors associated with stem cell-like properties, cell fate commitment and nervous system development including *bcl11ba*, *fgf8a*, *fgf20b*, *wnt2*, and *wnt3*. Several of these genes belong to Notch and Fgf signaling pathways which are known to play a role in supporting cell proliferation and HC differentiation (Jiang et al., 2014). The Fgf pathways are also involved in development, and downregulation of Fgf is required for proliferation of supporting cells and may inhibit transdifferentiation of supporting cells to HCs (Jacques et al., 2012a; Munnamalai and Fekete, 2013; Jiang et al., 2014; Maier and Whitfield, 2014). Conservation of these developmental pathways among vertebrate species may lend to the possibility of inducing HC regeneration in the mammalian cochlea using some of the novel ortholog targets identified as differentially expressed in zebrafish nsSCs.

## Materials and Methods

### RNA-seq Dataset Analysis

The raw data files from our published RNA-seq data set of adult zebrafish HCs and nsSCs (Barta et al., 2018) (NCBI SRP113243) were uploaded into CLC Genomics Workbench (CLC Bio, Waltham, MA, United States). Raw data files from zebrafish liver (Baumgart et al., 2016) (NCBI SRP033093) and microglia (Oosterhof et al., 2017) (NCBI SRP089875) were also imported. The combined dataset included three biological replicates of each cell type. The sequenced reads were aligned to the GRCz10 genome and read counts were normalized as RPKM. Statistical analysis was conducted in CLC Workbench, including two-way ANOVA FDR *p*-values. These values were exported as an Excel spreadsheet. Similarly, RNA-seq fastq files for adult mouse PCs, DCs, and inner and outer HCs (Li et al., 2018; Liu et al., 2018) (NCBI SRP133879 and SRP133880) were uploaded into CLC Workbench along with mouse liver (Fradejas-Villar et al., 2017) (NCBI SRP078005). The sequenced reads were aligned to the GRCm38 genome, read counts were normalized as RPKM values, statistical analysis was conducted, and all values were exported to Excel. The datasets included at least two to three biological replicates for each cell type. A list of all datasets and samples used can be found in Supplementary Table 1.

### Identification of Zebrafish and Mouse Protein-Coding Orthologs

A list of orthologous zebrafish-to-mouse genes was generated using the Ensembl Biomart web-based platform to extract information from the Ensembl Genes 91 database (Zerbino et al., 2018). A comparative genomics analysis, using zebrafish (*Danio rerio*) as the reference organism, generated a list of orthologous protein-coding mouse genes. After selecting the dataset zebrafish genes (GRCz10), the filter under gene type, protein-coding was selected. Additional attributes, within the homologs category, were selected to produce a list of mouse gene orthologs compared to the zebrafish. In addition to the gene stable ID and gene name, the type of homology to the reference gene, percent identity, WGA, and GOC were selected. The redundant zebrafish gene stable IDs were removed from the output file since they represented each transcript variant of the gene, thus producing a final list of 17,498 orthologs. This process was completed using the mouse (*Mus musculus*) as the reference organism for comparison.

The Ensembl Biomart ortholog quality controls are based on two pipelines that characterize the likelihood of orthology using different approaches to analyze genetic similarity (Zerbino et al., 2018). The gene order conservation (GOC) score defines orthologous relationships based on conservation of the two genes upstream and two genes downstream of the target gene in the reference and query species. Each gene match is given a score of 25 percent, the highest score being 100 percent which indicates a match of all four neighboring genes in both species. The whole genome alignment (WGA) score is based on the nucleotide sequence and assumes that true orthologs will have highly conserved sequences. The coverage of the aligned sequences, with a heavier weight given to exon rather than intron sequences, is used to generate a score (averaged for each alignment from the pair of genes) from 0 to 100 for the ortholog prediction. Ensembl defined the high-confidence orthology thresholds based on the most-recent common ancestor, in this case *Mammalia* (mouse and human) and *Percomorpha* (zebrafish), as the following minimums: GOC score ≥ 75 and/or WGA score ≥ 75, and percent identity ≥ 50. The UCSC Table Browser was used to download additional annotation data for the zebrafish and mouse protein-coding genes including chromosome location and number of transcript variants and exons (Karolchik et al., 2004).

### Biological Function Enrichment Analysis

In order to identify biological functions enriched in or unique to the supporting cell populations, a GO analysis was conducted. Differential gene expression analyses for each species were conducted using iDEP, while further functional analysis of high-confidence orthologs was conducted using ShinyGO (*p*-value < 0.05) (Ge et al., 2018). The Gene Ontology Consortium annotated GO groups was used for verification of gene enrichment categories (Ashburner et al., 2000; Gene Ontology Consortium, 2019). No custom code was used for analysis.

### Immunofluorescence

Adult zebrafish [10 months; *Tg(pou4f3:mGFP)*] were euthanized and decapitated, and the whole head was fixed in 4% paraformaldehyde (PFA) in 1X phosphate buffered saline (PBS) overnight. The auditory end organs (lagena, utricle, saccule, and semicircular canals) were removed from the skull capsule and placed in hydrogel overnight. Following tissue clearing and decalcification, the tissues were incubated in block-Triton overnight at 4°C. Primary antibodies (anti-Fgf8a: GTX12126 from Genetex, 1:250; anti-GFP: 600-101-215 from Rockland, 1:1000) were diluted in block-Triton and incubated overnight, then washed in 1XPBS-Triton (0.1%). Overnight incubation with secondary antibodies (Fgf8a: Goat anti-rabbit Alexa 633, A21071, Lot: 1387814, 1:2000; GFP: donkey anti-goat Alexa 488, A11055, Lot:1463163, 1:2000 both from Thermo Fisher) was followed with washes in 1XPBS-Triton (0.1%), refixation in 4% PFA-1X PBS, and finally 1X PBS wash. Samples were incubated in 50% glycerol/50% PBS at 4°C. To prepare for imaging, the whole tissue was placed in OCT embedding compound at 4°C for 24 h to allow full infiltration of tissue. The tissue was then placed on a metal disk, placed at an appropriate angle for sectioning, and rapidly frozen to −20°C. The tissue was cut into 5–10 μm slices and mounted on glass slides with antifade solution (Prolong Antifade Kit, Invitrogen, Carlsbad, CA, United States) before imaging on a Leica Confocal Microscope (Leica TCS SP8 MP). This study was carried out in accordance with the AAALAC International guidelines and the protocol was approved by the Creighton University IACUC.

## Results

### Classification of Zebrafish and Mouse Protein-Coding Orthologs

To compare protein-coding genes between zebrafish and mouse supporting cells, we first compiled a list of zebrafish and mouse protein-coding gene orthologs based on published, annotated genomes using the Ensembl Biomart database (Zerbino et al., 2018). An initial comparative genomics analysis revealed that 61.5% of mouse protein-coding genes have at least one zebrafish ortholog with 45% classified as zebrafish-to-mouse one-to-one orthologs, comparable to 47% zebrafish-to-human one-to-one orthologs (Howe et al., 2013) (Table 1). Conservation among the 9,900 one-to-one zebrafish-to-mouse orthologs suggests that many protein-coding genes among vertebrates encode proteins with similar functions (Altenhoff et al., 2012). Our study had a total of 25,098 protein-coding zebrafish genes while Howe et al. (2013) reported a total of 26,206; however, their comparative genomics analysis included a greater number of annotated genes than the Ensembl 91 database used for this analysis (Collins et al., 2012). Compared to mammals, zebrafish have a greater number of protein-coding genes and genes belonging to paralogous groups (many-to-one and many-to-many); therefore, a direct comparison of orthologous genes between zebrafish and mouse results in a single mammalian gene corresponding to many zebrafish genes. Genome duplications prior to and after the divergence of the species results in paralogous genes, which may or may not retain related functions, and in many cases, acquire new biological functions distinct from the ancestral gene (Postlethwait et al., 1998, 2000; Koonin, 2005). The ancestral gene of the paralogous group is more likely to encode a protein with sequence and functional homology most similar to that of its mammalian counterpart (Altenhoff et al., 2013). Redundant mouse genes corresponding to multiple zebrafish paralogs were removed from the analysis, resulting in a total of 13,557 protein-coding gene orthologs in mouse compared to 17,498 in zebrafish.

**TABLE 1:** Classification of orthologous relationships of zebrafish and mouse protein-coding genes.

**Gene Ortholog Relationship**	**Zebrafish (GRCz10)**	**Mouse (GRCm.38.5)**
One-to-one	9,900	9,900
Many-to-one	6,723	3,425
Many-to-many	911	232
Protein-coding gene ortholog total	17,498	13,557
Unique genes	7,600	8,501
Protein-coding gene total	25,098	22,058

*Protein-coding gene orthologs identified in a zebrafish-to-mouse whole-genome comparison based on the Ensembl Genes 91 database. Orthologs were classified as one-to-one, many-to-one, or many-to-many. Those without a corresponding gene ortholog in the other species were classified as unique genes.*

The protein-coding gene orthologs of both zebrafish and mouse are evenly distributed throughout the respective genomes and similar in the number of exons encoding the functional proteins (Supplementary Figure 1). Notably, the majority of zebrafish genes may encode four or fewer transcript variants while many mouse genes encode greater than eight variant transcripts, though it is likely that not all of these transcripts encode functional proteins. A GO analysis of the one-to-one orthologs indicated functional enrichment in highly conserved biological processes associated with general cellular functions such as biosynthesis and metabolic processes, gene expression, and development (Supplementary Figure 2). The gene orthologs classified in many-to-one and many-to-many categories also have conserved functions among vertebrates, though various paralogs of these genes in zebrafish and mouse may encode proteins with different functions. The many-to-one orthologs showed enrichment in biological processes encoded by greater molecular diversity such as cell signaling and membrane transport (Supplementary Figure 2).

### Characterization of Protein-Coding Gene Orthologs Expressed in Zebrafish and Mouse Inner Ear Supporting Cells

**FIGURE 1:**
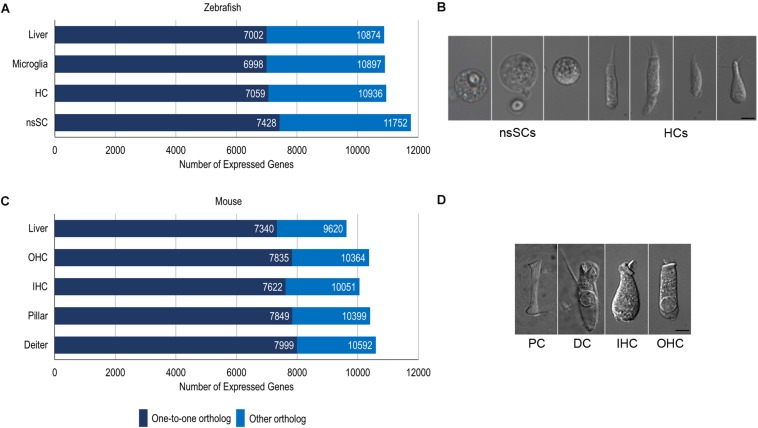
Cell type-specific gene expression in zebrafish and mouse. **(A)** The total number of expressed gene orthologs (RPKM ≥ 0.10, FDR-adjusted *p*-value ≤ 0.10) in zebrafish nsSCs, HCs, microglia, and liver. The number of expressed one-to-one orthologs out of the total number of classified orthologs is shown in dark blue. **(B)** Representative images of zebrafish nsSCs and HCs; Bar: 5 μm. **(C)** The total number of expressed gene orthologs (RPKM ≥ 0.10, FDR-adjusted *p*-value ≤ 0.10) in mouse DCs, PCs, IHCs, OHCs, and liver cells. **(D)** Representative image of a mouse PC, DC, IHC, and OHC; Scale bar: 5 μm (adapted from Liu et al., 2014).

We compared zebrafish nsSCs and mouse PCs and DCs to species-specific hair cells to reveal shared and unique biological characteristics among the vertebrate inner ear non-sensory cell populations using the cell-type specific gene expression data from our previous RNA-seq analyses (Barta et al., 2018; Li et al., 2018; Liu et al., 2018). Raw RNA-seq data sets from zebrafish and mouse (Supplementary Table 1) were mapped to each respective genome and expression values normalized as RPKM. The comprehensive merged list of protein-coding gene ortholog expression values is provided as a searchable Excel table (Supplementary Data Sheet 1). Of the total orthologs (17,498 and 13,557 for zebrafish and mouse respectively), 304 did not have expression data for the zebrafish and/or mouse cells, so they were excluded from the analysis. It should be noted that while an arbitrarily set expression cutoff of 0.1 RPKM (FDR *p*-value ≤ 0.10) was used for both species, the actual values are not quantitatively equivalent because they are derived from different species and thus cannot be normalized as a single dataset. Of the 17,228 protein-coding gene orthologs detected in the zebrafish dataset, a total of 11,752 and 10,936 genes were expressed above cutoff in zebrafish nsSCs and HCs, respectively (Figure 1A). The well-differentiated mouse PC and DC populations expressed 10,592 and 10,399 genes out of 13,495 total orthologs; while the IHCs expressed 10,051 and OHCs expressed 10,364 orthologous genes (Figure 1C). Representative images of zebrafish nsSCs and HCs, and a mouse PC, DC, IHC, and OHC are shown in Figures 1B,D, respectively.

### Conservation of Biological Functions of Differentially Expressed Genes in Non-sensory Supporting Cells

**FIGURE 2:**
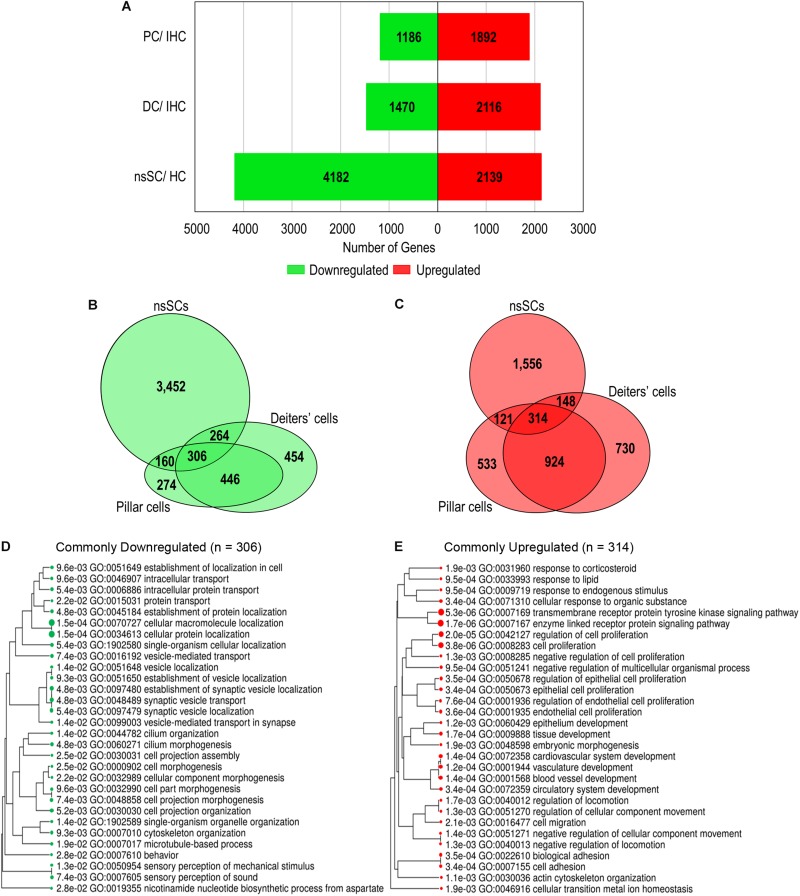
Differential gene expression among vertebrate inner ear supporting cells. Differentially expressed genes were classified as those expressed ≥ 0.10 RPKM in each cell type (FDR-adjusted *p*-value ≤ 0.10), with a log2 fold change greater than 1.0 or less than –1.0. **(A)** The total number of upregulated (red) and downregulated (green) genes in zebrafish nsSCs compared to zebrafish HCs, as well as mouse DCs and PCs compared to IHCs of the organ of Corti. **(B)** Venn diagram showing the number of shared and unique downregulated genes in zebrafish nsSCs compared to HCs, and mouse PCs or DCs compared to IHCs. The size of the circle is relative to the number of genes. **(C)** The shared and unique upregulated genes among the supporting cell populations. **(D)** GO analysis (ShinyGO) of enriched biological processes of commonly downregulated gene orthologs in zebrafish nsSCs and mouse PCs and DCs (*n* = 306). The hierarchal clustering tree shows related GO terms grouped together based on the number of common genes (FDR *p*-value ≤ 0.05). **(E)** GO enriched biological processes of the commonly upregulated genes (*n* = 314).

A gene expression analysis of zebrafish and mouse inner ear supporting cells illuminated both conserved and distinct biological properties, compared to species-specific HCs. We analyzed differentially expressed upregulated and downregulated genes in supporting cells with reference to species-specific HCs. Upregulated genes were identified as those with a log2 fold change of 1.0 or greater, while downregulated genes had a log2 fold change less than −1.0 (FDR *p*-value ≤ 0.10) (Supplementary Data Sheet 2). A total of 2,139 genes were upregulated in zebrafish nsSCs compared to HCs, while 4,182 were downregulated (Figure 2A). Conversely, the pattern observed in mouse inner ear cells showed a greater number of genes upregulated in supporting cells compared to IHCs. DCs had 2,116 upregulated and only 1,470 downregulated genes compared to IHCs, while PCs had 1,892 upregulated and 1,186 downregulated genes compared to IHCs.

A direct comparison of the commonly up and downregulated genes among nsSCs, PCs and DCs distinguished shared and distinct cellular phenotypes among these supporting cell populations. Among the three cell types, several genes were commonly up or downregulated (Figures 2B,C), indicating some conserved functions among both the HC and non-sensory supporting cell populations in vertebrate species. Closer examination of genes commonly downregulated among nsSCs, PCs, and DCs (*n* = 306) distinguished supporting cell function from the commonly derived HCs. A ShinyGO analysis identified biological processes including synaptic vesicle transport, cilium morphogenesis, cell projection assembly, and sensory perception of sound were significantly downregulated in supporting cell populations compared to HCs (Figure 2D). A similar analysis revealed that the commonly upregulated genes in supporting cells (*n* = 314) were enriched in biological processes associated with negative regulation of cell proliferation, development, and cell adhesion (Figure 2E).

**FIGURE 3:**
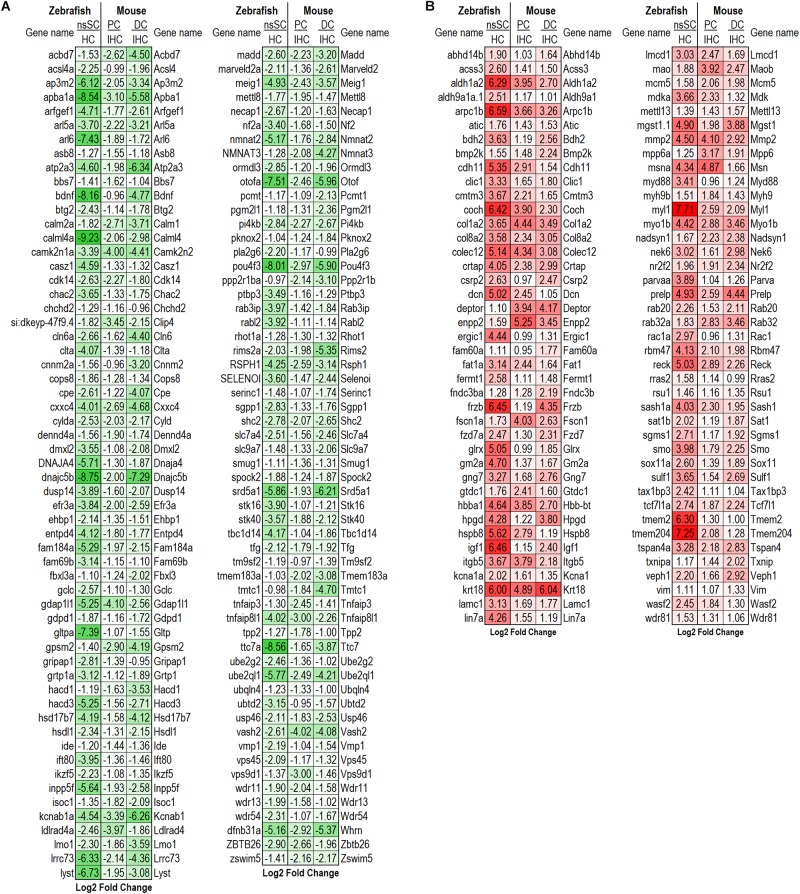
Differentially expressed high-confidence gene orthologs in all zebrafish and mouse supporting cells. **(A)** Log2 fold change values of the 118 high-confidence gene orthologs commonly downregulated in zebrafish nsSCs and mouse supporting cells (corresponding mouse gene ortholog name on the right.) **(B)** Log2 fold change values of the 82 high-confidence gene orthologs commonly upregulated in nsSCs and mouse supporting cells. High confidence orthologs: ≥50% ID, ≥75 WGA and/or ≥75 GOC (see Materials and Methods).

To further illuminate the highly conserved functions among the vertebrate cell populations, the common differentially expressed genes were reduced to the high-confidence orthologs. This orthologous relationship is defined based on conservation of the genes among zebrafish and mouse based on sequence-identity, whole-genome alignment, and gene order conservation, as compared to their most recent common ancestor (see Methods: ≥50% ID, ≥75 WGA and/or ≥75 GOC). Of the 306 commonly downregulated genes, 118 were high-confidence orthologs. The log2 fold change between ncSCs and HCs, as well as PCs or DCs and IHCs are shown (Figure 3A). Notable HC genes including *atp2a3* (*Atp2a3*), *btg2* (*Btg2*), *dnajc5b* (*Dnajc5b*), *otofa* (*Otof*), and *pou4f3* (*Pou4f3*) were downregulated as expected (Hu et al., 2009; Pan et al., 2012; Ku et al., 2014; Liu et al., 2014; Erickson and Nicolson, 2015; Michalski et al., 2017). Of the 314 commonly upregulated genes, 82 were identified as high-confidence orthologs (Figure 3B). High-confidence orthologs associated with cell proliferation (GO: 0008283) included *aldh1a2* (*Aldh1a2*), *col8a2* (*Col8a2*), *frzb* (*Frzb*), *fzd7a* (*Fzd7*), *hpgd* (*Hpgd*), *mmp2* (*Mmp2*), *nr2f2* (*Nr2f2*), *rac1a* (*Rac1*), *smo* (*Smo*), *sulf1* (*Sulf1*) and *tax1bp3* (*Tax1bp3*).

### Shared and Distinct Properties Between Zebrafish and Mouse Supporting Cell Populations

**FIGURE 4:**
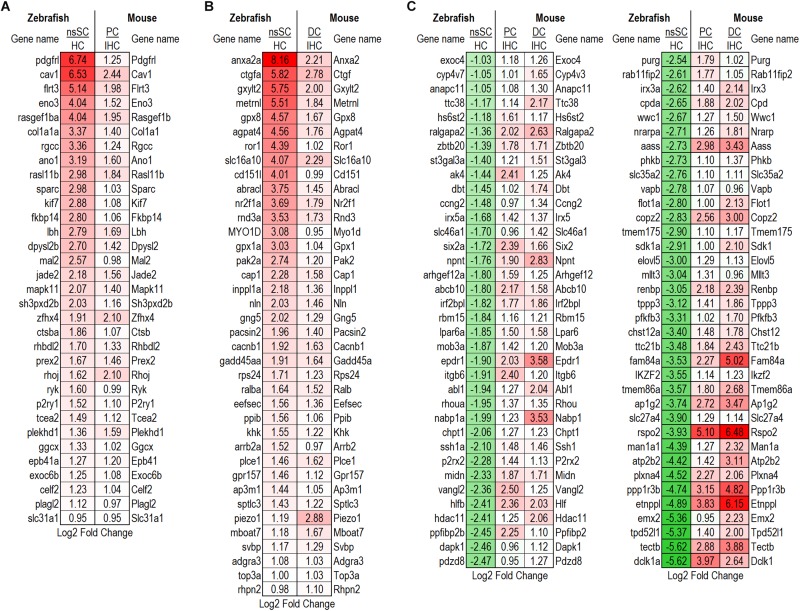
Differentially expressed high-confidence gene orthologs among supporting cells. **(A)** Log2 fold change values for the 33 high-confidence, out of the 121 total, gene orthologs commonly upregulated by zebrafish nsSCs and PCs. **(B)** Log2 fold change values for the 38 high-confidence, out of the 148 total, gene orthologs commonly upregulated by zebrafish nsSCs and DCs. **(C)** High-confidence orthologs downregulated in zebrafish nsSCs, compared to HCs, that were upregulated in mouse PCs or DCs compared to IHCs (*n* = 72).

Examination of shared characteristics among the cell populations showed that mouse PCs and DCs had 446 common downregulated genes while nsSCs shared 160 and 264 genes with PCs or DCs, respectively (Figures 2B,C). A similar relationship was observed among the commonly upregulated genes, though PCs and DCs shared more than twice the number of upregulated genes (*n* = 924) than downregulated genes. DCs and nsSCs shared a greater number of enriched genes (412 both up and downregulated) than PCs and nsSCs (281), suggesting that these cell populations are more similar phenotypically. This is consistent with the notion that PCs are a mammalian innovation and their gene expression profile is more similar to that of OHCs (Liu et al., 2018). Of the 121 upregulated PC and nsSC genes 33 were high-confidence orthologs, while 38 of the 148 upregulated genes in DC and nsSCs were high-confidence orthologs (Figures 4A,B). A ShinyGO analysis of the commonly upregulated genes in DCs and nsSCs indicated enrichment in biological processes of cell migration and locomotion, epithelium development and regulation of epithelial cell proliferation, neurogenesis, and cell/embryo development (Supplementary Data Sheet 3). This included high-confidence orthologs that play a role in epithelial cell proliferation (GO:0050673): *ccnd1* (*Ccnd1*), *fabp7b* (*Fabp7*), *ptprk* (*Ptprk*), and *sox11a* (*Sox11*), and epithelium development (GO:0060429): *gpx1a* (*Gpx1*), *ldb2b* (*Ldb2*), and *smad6b* (*Smad6*). Interestingly, several of the highly upregulated genes in both nsSCs and DCs play a role in neurogenesis (GO:0022008) including *abi1a* (*Abi1*), *nme2b.2* (*Nme2*), *nr2f1a* (*Nr2f1*), *pak2a* (*Pak2*), and *viml* (*Vim*), as well as regulation of multicellular organismal development (GO:2000026) *arrb2a* (*Arrb2*), *ctgfa* (*Ctgf*), *gng5* (*Gng5*), *igf1* (*Igf1*), *prickle1b* (*Prickle1*), and *nln* (*Nln*). However, PCs do not appear to share these intrinsic proliferative properties with nsSCs. Among the biological processes of genes upregulated in both nsSCs and PCs (*n* = 121) were actin cytoskeletal organization, cell–cell adhesion, and membrane organization (Supplementary Data Sheet 3). Several of these high-confidence ortholog genes also positively regulate cellular response to stimulus (GO: 0048584) *cav1* (*Cav1*), *col1a1a* (*Col1a1*), *rgcc* (*Rgcc*), *sfrp1b* (*Sfrp1*), and *wnt7ba* (*Wnt7b*).

Further evidence of the differential properties among mouse PCs and DCs, and zebrafish nsSCs was shown in the 942 genes upregulated only in PCs and DCs. Interestingly, 72 of these genes were high-confidence orthologs that were significantly downregulated in nsSCs compared to zebrafish HCs (Figure 4C). These genes showed enrichment in biological processes including regulation of the Wnt and Ras signaling pathways, tissue morphogenesis, and development (Supplementary Data Sheet 3).

### Functions of Uniquely Upregulated Gene Orthologs in Zebrafish nsSCs

**FIGURE 5:**
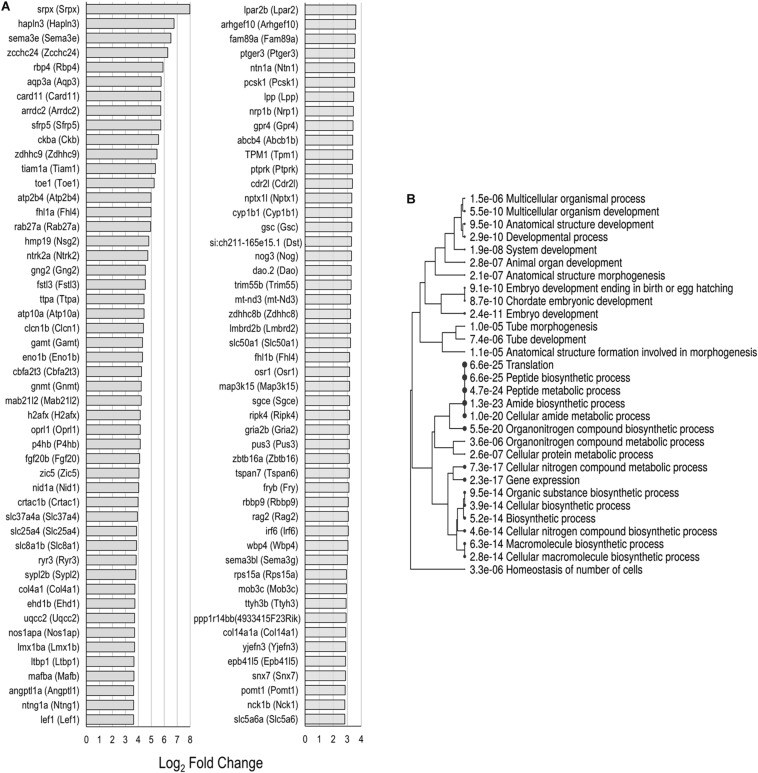
Unique upregulated genes in nsSCs. **(A)** Log2 fold change values of the top 50 high-confidence gene orthologs uniquely upregulated in zebrafish nsSCs (*n* = 386) compared to HCs. **(B)** ShinyGO analysis identified enriched biological processes of uniquely upregulated zebrafish nsSC genes.

Each of the supporting cell populations expressed a number of uniquely downregulated and upregulated genes including 274 and 533 in PCs, and 454 and 730 in DCs, respectively. The zebrafish nsSCs had the highest number of uniquely downregulated (3,452) and upregulated genes (1,556) when compared to mouse supporting cells (Figures 2B,C). A ShinyGO analysis of the 1,556 genes uniquely upregulated in zebrafish nsSCs showed enrichment in biological processes of embryological development, anatomical structure morphogenesis, homeostasis of number of cells in a tissue, gene expression, and translation (Supplementary Data Sheet 3). 386 genes were identified as high-confidence orthologs, with the top 50 expressed at a log2 fold change of 2.0 of higher in nsSCs compared to HCs (Figure 5A). A ShinyGO analysis revealed genes associated with anatomical structure morphogenesis (GO: 0009653): *dlx3b* (*Dlx3*), *irf6* (*Irf6*), *lef1* (*Lef1*), *mafba* (*Mafb*), *sema3e* (*Sema3e*), *sox7* (*Sox7*), and *zic5* (*Zic5*); embryo development (GO:0009790): *fgf20b* (*Fgf20*), *lmx1ba* (*Lmx1b*), *mkxa* (*Mkx*), *osr1* (*Osr1*), *sfrp5* (*Sfrp5*), and *yes1* (*Yes1*); and, homeostasis of number of cells (GO:0048873): *tal1* (*Tal1*), *lmo2* (*Lmo2*), *sae1* (*Sae1*), *rplp1* (Gm10073), and *rasa3* (*Rasa3*) (Figure 5B).

### Unique Expression of Gene Orthologs in Zebrafish nsSCs

**FIGURE 6:**
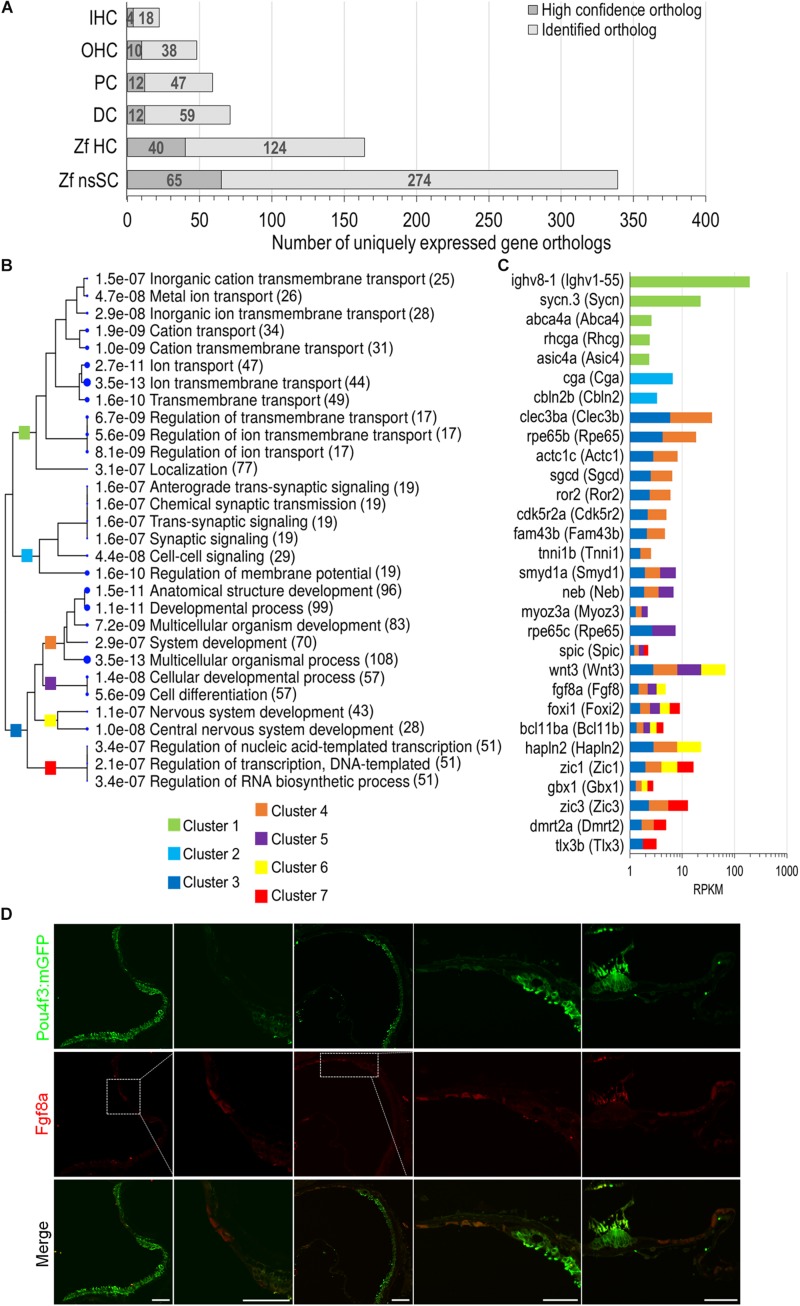
Unique expression of gene orthologs in nsSCs. **(A)** Total number of uniquely expressed protein-coding gene orthologs in zebrafish and mouse inner ear cells. The dark gray portion of the bar indicates the proportion of high-confidence orthologs, out of the total unique orthologs, that were uniquely expressed in each cell type (≥0.10 RPKM) while below cutoff expression in the five other cell types. **(B)** The most significantly enriched biological process categories identified in a ShinyGO analysis of 339 uniquely expressed nsSC ortholog genes. The clustering tree indicates relatedness among the biological processes. There are no gene redundancies across clusters 1, 2, and 3; however, clusters 4 through 7 include genes that are grouped in multiple GO categories. The number of genes is shown to the right of each category. **(C)** Expression values (RPKM) of 30 of the top expressed genes in nsSCs categorized in the GO analysis. The color of the bar indicates the corresponding GO cluster categorization from **(B)** for each gene. **(D)** Confocal images of the adult zebrafish [*Tg(pou4f3:mGFP)*] inner ear epithelia. Hair cells expressing GFP were labeled with anti-GFP antibody (green), while Fgf8a expressing nsSCs were labeled with anti-Fgf8a antibody (red). From left to right: utricle, magnified utricle, lagena, magnified lagena, and ampullae of semicircular canal. There was some non-specific staining of the stereocilia bundle by both secondary antibodies. Scale bar: 50 μm.

To further distinguish the nsSC population from the other inner ear cell types, an expression analysis was conducted to identify genes solely expressed in each cell type. The comparative expression analysis included zebrafish nsSCs and HCs, and mouse IHCs, OHCs, PCs, and DCs (Figure 6A). The number of uniquely expressed genes in the mammalian inner ear cells ranged from 71 to 22 genes. The zebrafish nsSCs had the greatest number of uniquely expressed genes (*n* = 339) compared to all cell types, followed by 164 in HCs. Of the 339 uniquely expressed genes in nsSCs, 65 were high-confidence orthologs, suggesting that these highly conserved genes may be performing unique functions in the nsSCs in the zebrafish inner ear. A ShinyGO analysis of biological functions of the 339 uniquely expressed genes in nsSCs showed enrichment in several categories associated with known supporting cell functions, including ion transport (Cluster 1) and synaptic signaling (Cluster 2) (Figure 6B). Significant gene enrichment was also observed in categories of developmental and regulation of gene expression processes (i.e., DNA-templated transcription) (Cluster 3), further supporting the inherent stemness retained by the nsSC population. For reference, some of the genes in each cluster are shown in Figure 6C. Additional divergence of Cluster 3 showed that several of the highly expressed genes in nsSCs function in pathways regulating cell differentiation and/or nervous system development and may act as regulators of transcription; notable genes include: *wnt3*, *fgf8a*, *foxi1*, *bcl11ba*, *zic1*, *glox1*, *zic3*, *dmrt2a*, and *tlx3b*. Immunofluorescence of the adult zebrafish inner ear confirmed expression of Fgf8a (Figure 6D) in the utricle and lagena, as well as the ampullae of the semicircular canal. The expression pattern of Fgf8a in the utricle and lagena seemed to be present in only some of the nsSCs in the epithelia, further suggesting the heterogeneity of the cell population surrounding HCs. In the developing zebrafish auditory epithelia, new HCs form on the periphery of the existing HC population; thus the location of the Fgf8a expressing cells is consistent with these findings (Brignull et al., 2009; Schuck and Smith, 2009; Abbas and Whitfield, 2010). The expression of Fgf8a in the ampullae was more widely distributed in the nsSCs, indicating that more of these cells may retain the capacity to form new HCs. Regeneration of vestibular hair cells occurs in non-mammalian and mammalian vertebrates through adulthood, albeit from different regenerative mechanisms.

### Expression of Transcription Factors Among Supporting Cells in the Vertebrate Inner Ear

To further characterize the regulatory mechanisms endogenous to the vertebrate supporting cell populations, a list of annotated genes involved in regulation of gene expression was generated using the Gene Ontology Consortium (2019), Ashburner et al. (2000), Riken TF Database (Kanamori et al., 2004) and TFCat (Fulton et al., 2009). The list of 2,801 genes have functions that include nucleic acid-templated transcription, regulatory region DNA binding, chromatin-mediated transcriptional regulation, positive/negative regulation of transcription, regulation of RNA polymerase activity, nuclear binding activity, or other related functions. For the purposes of this paper, we will collectively refer to these genes as “transcription factors.” The complete list of transcription factor gene ortholog expression values is included as Supplementary Data Sheet 4.

A ShinyGO analysis of uniquely expressed nsSC genes identified 51 nucleic-acid templated transcription factors (Figure 6B, Cluster 7). A total of 24 genes involved in regulating gene expression were expressed only in nsSCs (*p*-value ≤ 0.10) including *bcn2*, *mdfi*, *nkx2.2a*, *foxi3b*, *barhl2*, *eomesa*, *neurog1*, *six3a*, and *gli1* (Supplementary Figure 3). Comparably, there were 14 transcription factor genes that were commonly expressed in zebrafish nsSCs and microglia (excluded from unique ortholog expression analysis) (Supplementary Figure 3). The nsSCs perform many functions analogous to the glia in the nervous system; therefore, these similar gene expression patterns are expected among the cell populations (Monzack and Cunningham, 2013). Some of these commonly expressed genes regulate cell fate specification and differentiation (*barhl2*, *eomesa, foxi3b*, *nkx6.2*, and *six3a*), as well as neurogenesis and neuron development (*nkx2.2a, npas4a*).

**FIGURE 7:**
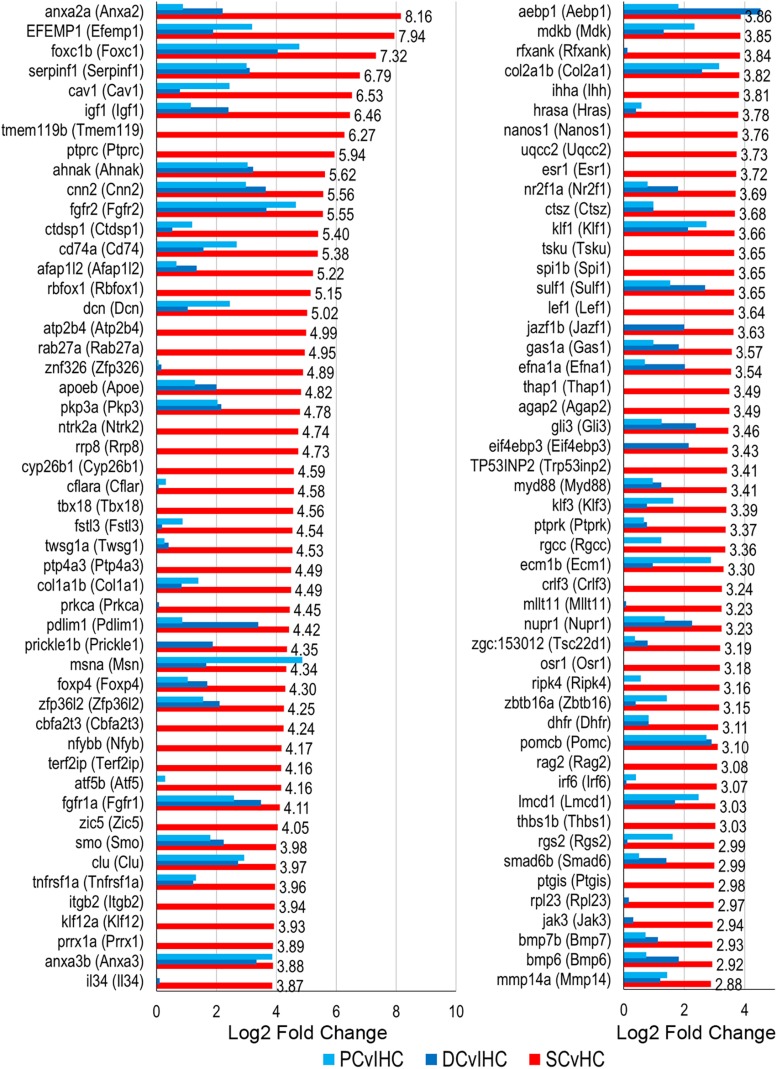
Transcription factor gene expression among adult zebrafish nsSCs. Log2 fold change values of the top 100 (*n* = 236) transcription factor genes upregulated in nsSCs compared to HCs (FDR-adjusted *p*-value ≤ 0.05, log2 fold change ≥ 1.0) (corresponding mouse gene ortholog name in parentheses). For comparison, fold changes for genes that are also upregulated in mouse PCs and DCs, compared to IHCs, are shown in blue.

We examined expressed, as well as up and downregulated transcription factors among vertebrate supporting cells to further elucidate their phenotypic identities and underlying regenerative properties. Transcription factors are generally expressed at lower levels compared to other protein coding genes and minimally expressed transcription factors can act as regulators of gene activation pathways inducing an exponential cellular response (Vaquerizas et al., 2009). The top 100 of the 321 transcription factor genes upregulated in zebrafish nsSCs compared to HCs are shown in Figure 7 (FDR-adjusted *p*-value ≤ 0.05, log2 fold change ≥ 1.0). The most highly upregulated transcription factor in nsSCs was *anxa2a* with a log2 fold change of 8.16, followed by *efemp1*, *foxc1b*, *serpinfi*, *cav1*, and *igf1*. Additionally, 70 of the 321 transcription factors were also significantly upregulated in both mouse PCs and DCs compared to IHCs, almost half of which were in the top 100.

**FIGURE 8:**
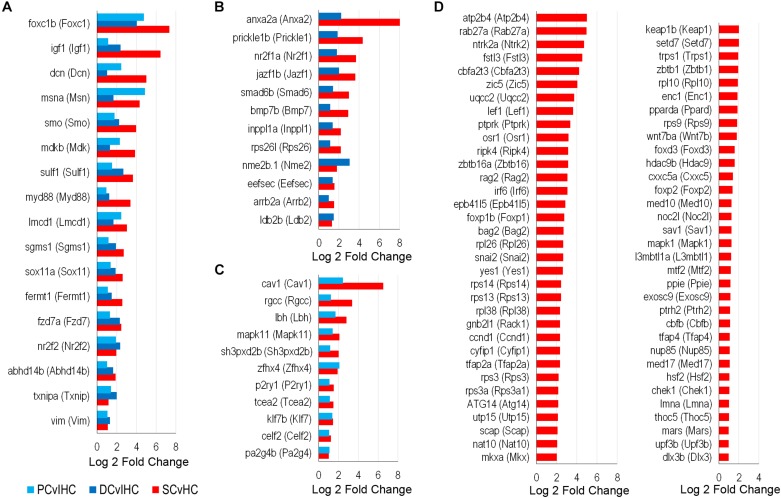
Transcription factors classified as high-confidence orthologs upregulated among the supporting cell populations. **(A)** Transcription factors upregulated (FDR-adjusted *p*-value ≤ 0.05, log2 fold change ≥ 1.0) in zebrafish nsSCs and mouse PCs and DCs (corresponding mouse gene ortholog name in parentheses). Log2 fold change values of zebrafish nsSCs compared to HCs, and mouse PCs or DCs compared to IHCs are shown. Commonly upregulated transcription factors in nsSCs and DCs **(B)** or PCs **(C)**. **(D)** The 67 high-confidence gene orthologs encoding transcription factors that are uniquely upregulated in zebrafish nsSCs.

To identify possible conserved regulatory processes in the supporting cell populations, the list of upregulated transcription factors was reduced to the 107 high-confidence orthologs. Seventeen of them were upregulated in nsSCs, as well as DCs and PCs: *foxc1b* (*Foxc1*), *msna* (*Msn*), *smo* (*Smo*), *sox11a* (*Sox11*), *fzd7a* (*Fzd7*), *nr2f2* (*Nr2f2*), and *vim* (*Vim1*) (Figure 8A). Upregulated transcription factors in both nsSCs and DCs (*n* = 12) included *anxa2a* (*Anxa2*), *prickle1b* (*Prickle1*), *nr2f1a* (*Nr2f1*), and *arrb2a* (*Arrb2*) (Figure 8B). Commonly upregulated transcription factors in nsSCs and PCs (*n* = 11) included *rgcc* (*Rgcc*), *lbh* (*Lbh*), *zfhx4* (*Zfhx4*), and *klf7b* (*Klf7*) (Figure 8C). There were 67 high-confidence orthologs that were upregulated only in nsSCs (Figure 8D). Several of these genes regulate anatomical structure development and morphogenesis, including: *cbfb*, *foxd3*, *irf6*, *lef1, mapk1*, *mkxa*, *osr1*, *tfap2a*, and *zic5*. Functional studies of some of these transcriptional regulators may reveal more of the underlying regenerative properties shared by, and unique to, these supporting cell populations.

### Deafness-Associated Gene Ortholog Expression in Supporting Cells

Expression of deafness genes has been observed in various supporting cells, in addition to hair cells, in the mammalian cochlea (Hilgert et al., 2010; Nishio et al., 2015; Li et al., 2018; Liu et al., 2018). A list of the protein-coding gene orthologs known to be associated with deafness phenotypes is presented in Supplementary Data Sheet 5. Several high-confidence orthologs were highly expressed in zebrafish nsSCs, and mouse PCs and DCs including *eya1* (*Eya1*), *gpsm2* (*Gpsm2*), *msrb3* (*Msrb3*), *prps1a* (*Prps1*), *slpr2* (*Slpr2*), *six1b* (*Six1*), and *wbp2* (*Wbp2*). Both *coch* (*Coch*) and *myh9b* (*Myh9*) were upregulated in all supporting cells compared to species-specific hair cells. Other highly expressed genes in the supporting cells included *aifm1* (*Aifm1*), *cd164* (*Cd164*), *clpp* (*Clpp*), *col2a1b* (*Col2a1*), *col9a2* (*Col9a2*), *hars* (*Hars2*), *kars* (*Kars*), and *tjp2b* (*Tjb2*). These genes are not expected to play an essential role in HC regeneration. However, loss or gain of function mutations in these genes may disrupt the supporting cell’s anchoring, glial, or transport functions, which may affect HC survival.

## Discussion

Various cellular mechanisms contribute to HC regeneration in the inner ear throughout the life of non-mammalian vertebrates, including transdifferentiation of supporting cells followed by division of resident stem cells. In order to elucidate the conservation and divergence of function among vertebrate supporting cell populations, we compared the transcriptome of zebrafish nsSCs to mouse PCs and DCs. This study was limited to protein-coding genes; however, there are other molecules, such as miRNAs, that are important in regulating gene expression during inner ear development and defining subsequent phenotypes of these distinct cell populations (Soukup et al., 2009; Lush and Piotrowski, 2014). A total of 2,139 genes were upregulated in zebrafish nsSCs compared to HCs, while almost twice as many genes (*n* = 4,182) were downregulated. Compared to mouse IHCs, DCs had 2,116 up and 1,470 downregulated genes, while PCs had 1,892 and 1,186 up and downregulated genes, respectively. Among the supporting cell populations there were around 300 commonly up and downregulated genes, compared to species-specific HC populations. As expected, the downregulated genes were associated with HC functions, while the upregulated genes were associated with various functions including cell adhesion, epithelium development and cell signaling pathways. All three supporting cells (nsSCs, DCs, and PCs) showed enrichment in genes involved in regulating epithelial cell proliferation and tissue development. Genes expressed only in DCs and nsSCs showed further enrichment in processes of epithelial proliferation, neurogenesis, and development. PCs and nsSCs showed enrichment in genes associated with cytoskeletal organization and cell–cell adhesion. These findings provide further evidence that mammalian supporting cells have variable conserved properties with zebrafish nsSCs. Previous transcriptome analyses suggest that while both PCs and DCs express genes that encode hair cell-specialization associated proteins, they appear to differ in their potential to proliferate and differentiate into HCs (Liu et al., 2018). The unique pattern of upregulated genes in mammalian supporting cells, compared to zebrafish nsSCs provides an explanation for their inherent loss of proliferative capacity.

A significant number of genes were uniquely downregulated and upregulated in the zebrafish nsSCs. This difference can partially be attributed to the higher number of orthologous genes in zebrafish. However, the considerable number of downregulated genes in nsSCs may also be attributed to the relatively undifferentiated nsSCs compared to the fully differentiated and morphologically distinct sensory HCs and mammalian supporting cells. The top uniquely downregulated genes in nsSCs included: *erich3, cib3, atp8a1, skorla, odf3l2, plcd4, klhl26, apt1b2b, saxo2, emb, and cacnb3b*. Several genes previously shown to be highly expressed in either PCs or DCs were also downregulated in nsSCs, including *bmp2a* (*Bmp2*), *dnajc5b* (*Dnajc5b*), *slc1a3a* (*Slc1a3*), *slc17a8* (Slc17a8) and *bmp4* (*Bmp4*), *cplx1* (*Cplxl*), *myo6a* (*Myo6*), *optn* (*Optn*), *qpct* (*Qpct*), respectively (Liu et al., 2018).

The top uniquely upregulated genes in nsSCs included: *srpx*, *hapln3*, *sema3e*, *zcchc24*, *rbp4*, *aqp3a*, *card11*, *arrdc2*, *sfrp5*, and *ckba*. Interestingly, *srpx* (*Srpx*), the most highly, uniquely upregulated gene in nsSCs, is classified in the GO category for negative regulation of cell proliferation involved in contact inhibition and has been well documented as a suppressor gene downregulated in several cancer cell lines (Yamashita et al., 1999). These findings suggest that many of the highly upregulated genes in the zebrafish nsSCs maintain more stem cell-like proliferative qualities, thus distinguishing them from the highly differentiated mammalian counterpart.

Several genes uniquely expressed in nsSCs are known to regulate cell fate commitment/determination, including *wnt3*, *foxi1*, and *fgf8a*. Identification of the up and downstream targets of these molecules may reveal regulatory mechanisms that directly or indirectly initiate nsSC proliferation and/or transdifferentiation into hair cells. Both Notch and Wnt signaling play an important role in proliferation of progenitor cells and cell fate determination in the vertebrate inner ear, specifying the identity and organization of sensory HCs and supporting cells (Chai et al., 2012; Jacques et al., 2012b; Mizutari et al., 2013; Jansson et al., 2015; Mittal et al., 2017). The inhibition of Notch signaling in the mammalian cochlea causes proliferation of supporting cells (Li et al., 2015). A recent study of zebrafish lateral line neuromasts identified a *wnt2* expressing, sub-population of supporting cells that resembled a self-renewing stem cell population (Jacques et al., 2014; Romero-Carvajal et al., 2015; Kniss et al., 2016; Lush et al., 2019). Interestingly, *wnt2* (*Wnt2*) was expressed at very high levels in nsSCs (244.6 RPKM), though it was excluded from the list of uniquely expressed genes because it was expressed above background in DCs (0.17 RPKM). The expression of *wnt2* and *wnt3* in nsSCs is consistent with the regeneration model in which some nsSCs will first proliferate/self-renew and then differentiate, rather than undergo direct transdifferentiation that may not require Wnt signaling (Li et al., 2015). Wnt signaling also intersects with calcium signaling pathways, driving cell migration and cell fate (Gómez-Orte et al., 2013). While *fgf8a* has been shown to be expressed early during otic induction and plays a role in differentiation of supporting cells, the observed expression in the adult zebrafish nsSCs, in the periphery of the auditory epithelia, suggests that these cells may retain some progenitor cell-like properties (Pirvola et al., 2002; Shim et al., 2005; Jahan et al., 2010). Additionally, *fgf20b*, while not uniquely but highly expressed in nsSCs (61.7 RPKM) compared to HCs (3.6 RPKM), acts downstream of Notch in prosensory induction and represses regenerative proliferation in the avian inner ear (Munnamalai et al., 2012; Ku et al., 2014). Fgf signaling inhibits transdifferentiation of supporting cells and its downregulation is necessary to induce proliferation of nsSCs (Jacques et al., 2012a; Monzack and Cunningham, 2013; Ku et al., 2014). The expression of these progenitor cell markers in adult zebrafish may help to identify specific nsSC subpopulations in epithelia of the inner ear for further examination. The specific roles of these Fgf expressing nsSCs in hair cell regeneration will need to be further explored; however, it is likely that downregulation of *fgf8a* and/or *fgf20b* may stimulate transdifferentiation of the nsSCs to a sensory cell fate (Maier and Whitfield, 2014). Additionally, variable expression of Fgf receptors in non-mammalian and mammalian inner ear cells may also account for differing proliferative responses resulting from cell-cell interactions, including cell fate determination (Mansour et al., 2013).

The unique expression of transcription factor genes among the nsSCs can also be informative of the intrinsic properties that are retained by this poorly understood cell population in the zebrafish inner ear. For example, several of these transcription factors function in biological pathways, including neurogenesis, cell proliferation and differentiation, maintenance of stem cell properties, and development. Evidence suggests that Zic1 and Zic3 enhance Notch signaling, inhibiting neuronal cell differentiation and thus maintaining proliferation of progenitor cell populations in the forebrain and retina (Inoue et al., 2007; Watabe et al., 2011). Zic proteins can also function as cofactors in Gli-regulated expression by altering transcriptional activation, and act on downstream targets involved in hair cell development and differentiation including Math1/Atoh1, Hes1, and Sox2 (Aruga et al., 1994; Aruga, 2004). Other genes of note include *foxi1* which plays an important role in the transcription of genes associated with inner ear function (Smith, 1998; Enerback et al., 2018), and the *foxi3* transcription factor is necessary for inducing gene expression for otic placode induction (Khatri et al., 2014; Birol et al., 2016). Additionally, Foxi1 and Foxi3b activate Jag1, which regulates Notch signaling and cell fate determination, and may also play a role in patterning of the inner ear epithelia (Hulander et al., 2003; Janicke et al., 2007). Additionally, functional studies of *bcl11ba* (*Bcl11b*) have shown that it can induce cellular quiescence to maintain progenitor cell populations and regulate *Notch* expression during differentiation, as well as regulate differentiation of postmitotic neurons (Simon et al., 2012; Zhang et al., 2012; Cai et al., 2017). The intersection of the above signaling pathways and others that regulate HC and supporting cell proliferation, such as cyclin dependent kinase inhibitors, is apparent in the many developmental and regeneration studies that have been conducted in non-mammalian and mammalian species. The shared properties between the zebrafish nsSCs, PCs and DCs revealed that while mammalian supporting cells can be directly targeted for transdifferentiation into HCs, the supporting cell populations should also be targeted for self-renewal and proliferation to maintain supporting cell populations in the organ of Corti.

The gene ortholog expression patterns observed in this analysis provide important clues about the similarities and differences between non-mammalian and mammalian supporting cells and reveal new molecular targets for regeneration of functional HCs and proliferation of supporting cells in the mammalian inner ear. We note that unlike the supporting cells in the mammalian organ of Corti, which are highly differentiated and have distinct morphologies, the nsSCs in the zebrafish auditory epithelium are heterogenous and lacking distinct morphological features. The population of nsSCs have not been well characterized; however, our immunostaining study showed distinctive Fgf8a-positive subpopulations on the periphery of the auditory epithelia (Figure 6D), a property of stem cells, supporting recent observations that Fgf signaling help regulate HC regeneration (Lush et al., 2019). The diversity among nsSCs in the zebrafish inner ear does not appear to be present in the sensory lateral line neuromasts. While HCs that populate the neuromast are similar to the HCs of the zebrafish inner ear, the nsSCs are classified into two distinctive populations; the inner supporting cells, and the mantle cells that form a ring encircling the neuromast (Lush and Piotrowski, 2014). Several studies of lateral line hair cell regeneration have shown that induced proliferation and differentiation of the neuromast inner supporting cells produces new HCs (Lopez-Schier and Hudspeth, 2006; Ma et al., 2008; Mackenzie and Raible, 2012; Lush et al., 2019). Conversely, in the zebrafish inner ear, regeneration of HCs occurs as a result of direct transdifferentiation of supporting cells into HCs without cell division (Millimaki et al., 2010). We speculate that the regenerative properties in the zebrafish inner ear are likely due to distinct subpopulations of supporting cells, that can be converted directly to HCs, while others retain their proliferative capacity as resident stem cells. Additionally, it is highly likely that there are also immune, glial, and neuronal cells interspersed throughout the epithelium that support HC function similar to the mammalian cochlea. Further characterization of these subpopulations using scRNA-seq will likely reveal distinctive transcriptomic signatures of the cell population surrounding the HCs of the inner ear epithelia.

## Data Availability Statement

All datasets generated for this study are included in the manuscript/Supplementary Files.

## Ethics Statement

The animal study was reviewed and approved by the Creighton University IACUC.

## Author Contributions

KG performed the analysis and wrote the manuscript. KG and HL performed the ICC experiments. KG, KK, and DH revised and finalized the manuscript.

## Conflict of Interest

The authors declare that the research was conducted in the absence of any commercial or financial relationships that could be construed as a potential conflict of interest.

## Abbreviations

DC: Deiters’ cell
FDR: false discovery rate
GO: gene ontology
GOC: gene order conservation score
HC: hair cell
IHC: inner hair cell
nsSC: non-sensory surrounding cell
OHC: outer hair cell
PC: pillar cell
RPKM: reads per kilobase per million mapped reads
WGA: whole genome alignment score.
