# Supplementary material for: Expression of Protein-Coding Gene Orthologs in Zebrafish and Mouse Inner Ear Non-sensory Supporting Cells
Source: Front Neurosci. 2019 Oct 18;13:1117. doi: 10.3389/fnins.2019.01117 (PMC6813431; doi:10.3389/fnins.2019.01117)
Supplement: Supplementary file 1 [file Table_1.pdf]

Supplement Table 1. RNA-seq Datasets

Barta, C. L., Liu, H., Chen, L., Giffen, K. P., Li, Y., Kramer, K. L., et al. (2018). RNA-seq transcriptomic analysis of adult zebrafish inner ear hair cells. *Scientific Data, 5*, 180005. *NCBI Sequence Read Archive* SRP113243. doi: 10.1038/sdata.2018.5

Sample	SRA Accession ID	GEO Sample ID	Cell Type	Organism
Zf nsSC 1	SRX3022434	GSM2712282	GFP-neg Control surrounding cells 1	Danio rerio
Zf nsSC 2	SRX3022435	GSM2712283	GFP-neg Control surrounding cells 2	Danio rerio
Zf nsSC 3	SRX3022436	GSM2712284	GFP-neg Control surrounding cells 3	Danio rerio
Zf HC 1	SRX3022431	GSM2712279	GFP+ Zebrafish Hair Cells 1	Danio rerio
Zf HC 2	SRX3022432	GSM2712280	GFP+ Zebrafish Hair Cells 2	Danio rerio
Zf HC 3	SRX3022433	GSM2712281	GFP+ Zebrafish Hair Cells 3	Danio rerio

Baumgart, M., Priebe, S., Groth, M., Hartmann, N., Menzel, U., Pandolfini, L., et al. (2016). Longitudinal RNA-seq analysis of vertebrate aging identifies mitochondrial complex I as a small-molecule-sensitive modifier of lifespan. *Cell Systems, 2*(2), 122-132. *NCBI Sequence Read Archive* SRP033093. doi: 10.1016/j.cels.2016.01.014

Sample	SRA Accession ID	GEO Sample ID	Cell Type	Organism
Zf Liver 1	SRX893410	GSM1620956	liver182 12m control 8w rep1	Danio rerio
Zf Liver 2	SRX893411	GSM1620957	liver183 12m control 8w rep2	Danio rerio
Zf Liver 3	SRX893412	GSM1620958	liver184 12m control 8w rep3	Danio rerio

Oosterhof, N., Holtman, I. R., Kuil, L. E., van der Linde, H. C., Boddeke, E. W., Eggen, B. J., and van Ham, T. J. (2017). Identification of a conserved and acute neurodegeneration-specific microglial transcriptome in the zebrafish. *Glia, 65*(1), 138-149. *NCBI Sequence Read Archive* SRP089875. doi: 10.1002/glia.23083

Sample	SRA Accession ID	GEO Sample ID	Cell Type	Organism
Zf Microglia 1	SRX2163312	GSM2310340	Microglia control (Sample 1)	Danio rerio
Zf Microglia 2	SRX2163316	GSM2310344	Microglia control (Sample 5)	Danio rerio
Zf Microglia 3	SRX2163320	GSM2310348	Microglia control (Sample 9)	Danio rerio

Liu, H., Chen, L., Giffen, K. P., Stringham, S. T., Li, Y., Judge, P. D., et al. (2018). Cell-specific transcriptome analysis shows that adult pillar and deiters' cells express genes encoding machinery for specializations of cochlear hair cells. *Frontiers in Molecular Neuroscience, 11*, 356. *NCBI Sequence Read Archive* SRP133879. doi: 10.3389/fnmol.2018.00356

Sample	SRA Accession ID	GEO Sample ID	Cell Type	Organism
Mouse Deiters 1	SRX3757318	GSM3028929	Deiters_1	Mus musculus
Mouse Deiters 2	SRX3757320	GSM3028930	Deiters_2	Mus musculus
Mouse Deiters 3	SRX3757321	GSM3028931	Deiters_3	Mus musculus
Mouse Deiters 4	SRX3757322	GSM3028932	Deiters_4	Mus musculus
Mouse Deiters 5	SRX3757323	GSM3028933	Deiters_5	Mus musculus
Mouse Deiters 6	SRX3757324	GSM3028934	Deiters_6	Mus musculus
Mouse Pillar 1	SRX3757325	GSM3028935	Pillar_1	Mus musculus
Mouse Pillar 2	SRX3757326	GSM3028936	Pillar_2	Mus musculus
Mouse Pillar 3	SRX3757327	GSM3028937	Pillar_3	Mus musculus
Mouse Pillar 4	SRX3757328	GSM3028938	Pillar_4	Mus musculus
Mouse Pillar 5	SRX3757329	GSM3028939	Pillar_5	Mus musculus
Mouse Pillar 6	SRX3757330	GSM3028940	Pillar_6	Mus musculus

Li, Y., Liu, H., Giffen, K. P., Chen, L., Beisel, K. W., and He, D. Z. Z. (2018). Transcriptomes of cochlear inner and outer hair cells from adult mice. *Scientific Data, 5*, 180199. *NCBI Sequence Read Archive* SRP133880. doi: 10.1038/sdata.2018.199

Sample	SRA Accession ID	GEO Sample ID	Cell Type	Organism
Mouse IHC 1	SRX3757335	GSM3028941	IHC_1	Mus musculus
Mouse IHC 2	SRX3757336	GSM3028942	IHC_2	Mus musculus
Mouse IHC 3	SRX3757337	GSM3028943	IHC_3	Mus musculus
Mouse IHC 4	SRX3757338	GSM3028944	IHC_4	Mus musculus
Mouse OHC 1	SRX3757339	GSM3028945	OHC_1	Mus musculus
Mouse OHC 2	SRX3757340	GSM3028946	OHC_2	Mus musculus
Mouse OHC 3	SRX3757341	GSM3028947	OHC_3	Mus musculus
Mouse OHC 4	SRX3757343	GSM3028948	OHC_4	Mus musculus
Mouse OHC 5	SRX3757344	GSM3028949	OHC_5	Mus musculus
Mouse OHC 6	SRX3757345	GSM3028950	OHC_6	Mus musculus

Fradejas-Villar, N., Seeher, S., Anderson, C. B., Doengi, M., Carlson, B. A., Hatfield, D. L., et al. (2017). The RNA-binding protein Secisbp2 differentially modulates UGA codon reassignment and RNA decay. *Nucleic Acids Research, 45*(7), 4094-4107. *NCBI Sequence Read Archive* SRP078005. doi: 10.1093/nar/gkw1255

Sample	SRA Accession ID	GEO Sample ID	Cell Type	Organism
Mouse Liver 1	SRX1900397	GSM2227376	Secisbp2 wild type liver Rep1	Mus musculus
Mouse Liver 2	SRX1900398	GSM2227377	Secisbp2 wild type liver Rep2	Mus musculus
Mouse Liver 3	SRX1900401	GSM2227380	Trsp wild type liver Rep1	Mus musculus
Mouse Liver 4	SRX1900402	GSM2227381	Trsp wild type liver Rep2	Mus musculus
