# Supplementary figures and images for: Expression of Protein-Coding Gene Orthologs in Zebrafish and Mouse Inner Ear Non-sensory Supporting Cells
Source: Front Neurosci. 2019 Oct 18;13:1117. doi: 10.3389/fnins.2019.01117 (PMC6813431; doi:10.3389/fnins.2019.01117)

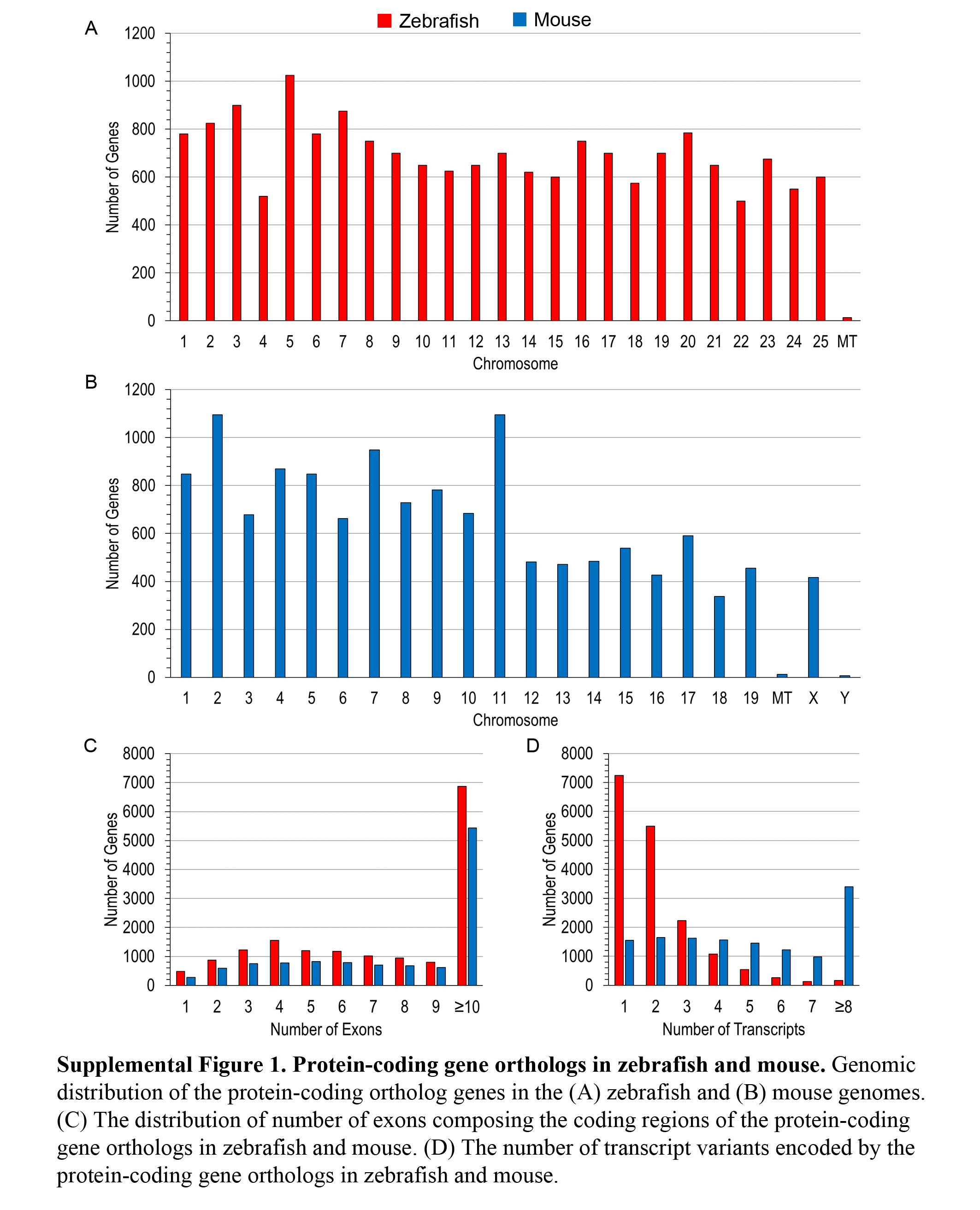

Supplement: Supplementary file 2 [file Image_1.TIF]

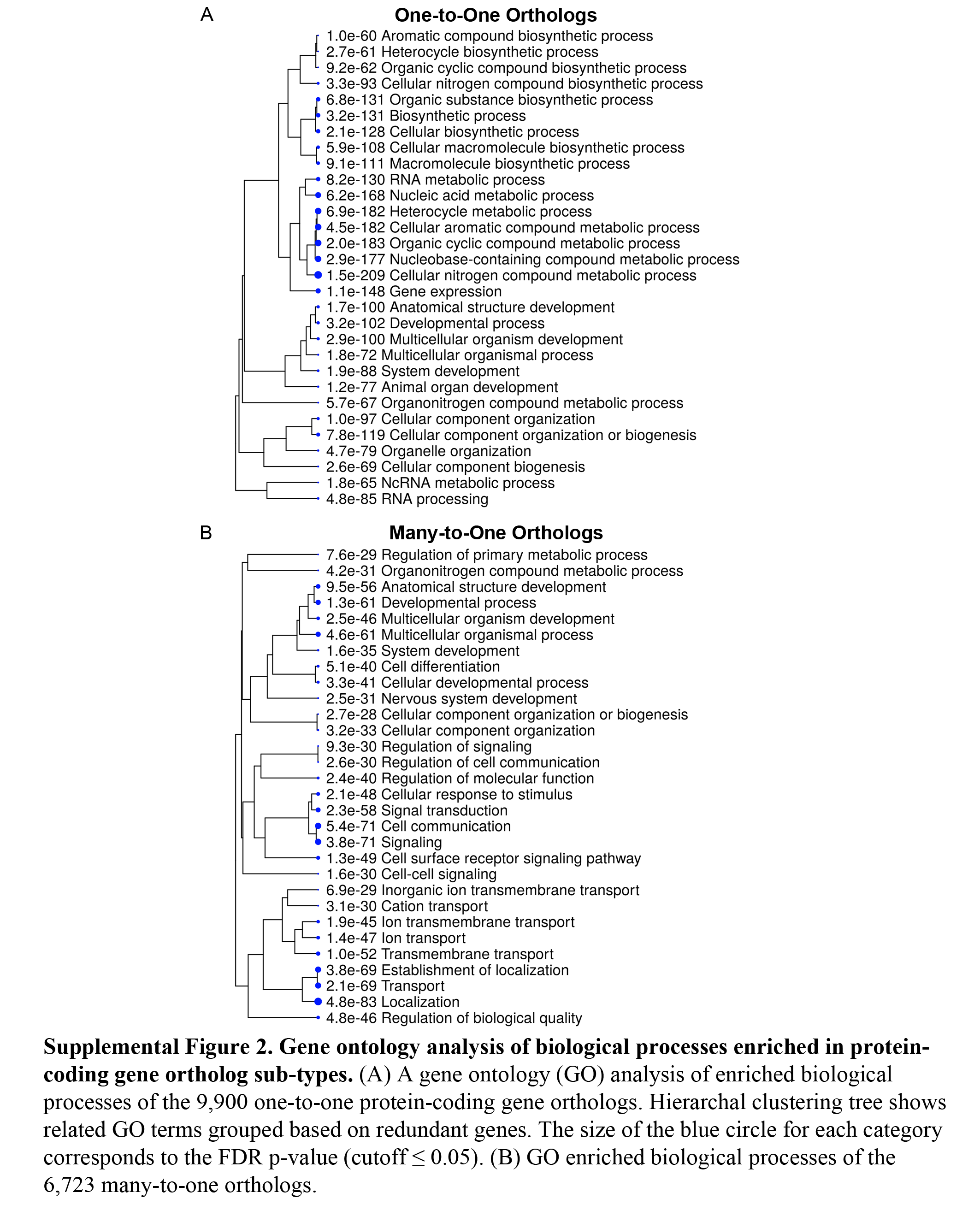

Supplement: Supplementary file 3 [file Image_2.TIF]

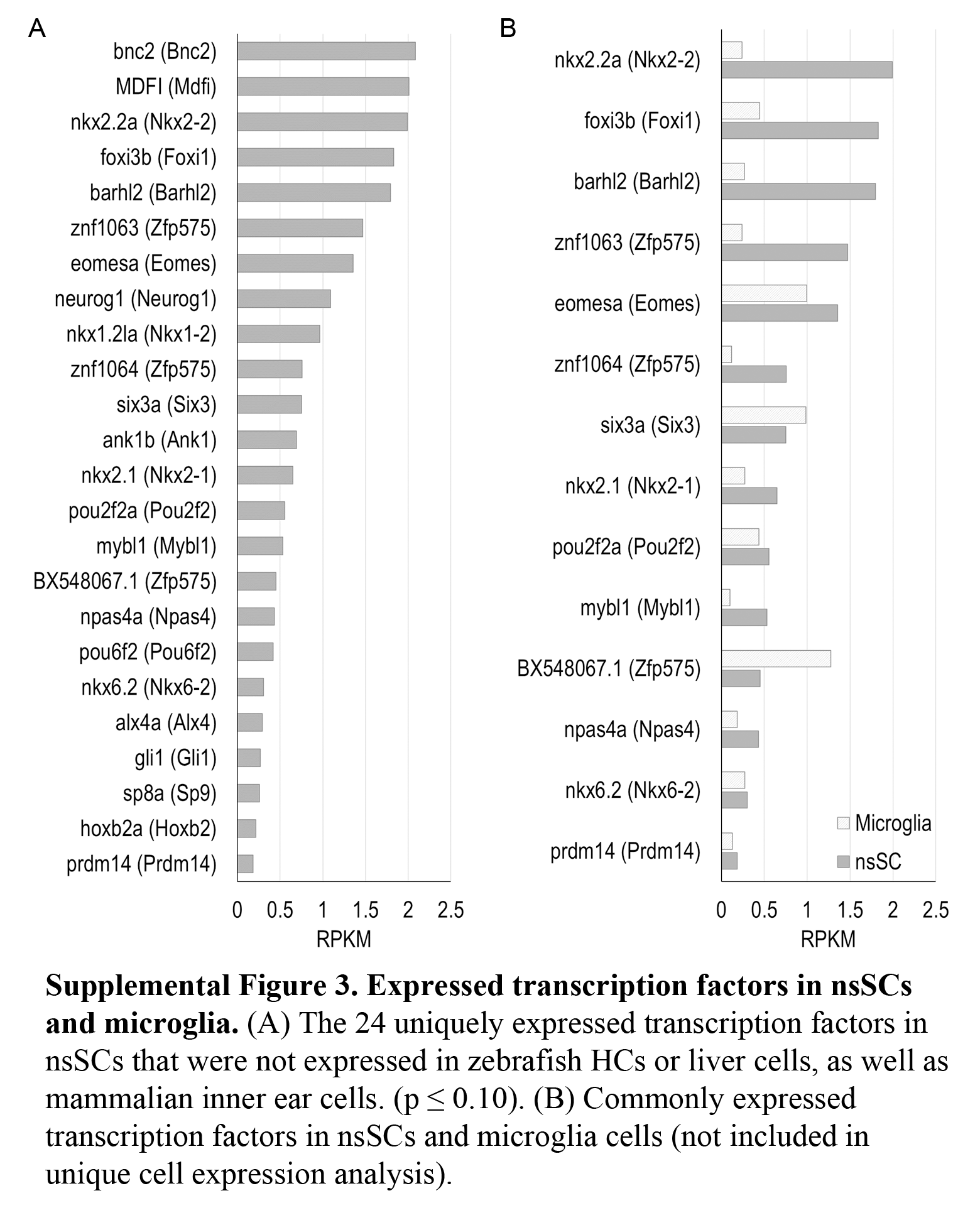

Supplement: Supplementary file 4 [file Image_3.TIF]
