# Supplementary material for: Expression of Protein-Coding Gene Orthologs in Zebrafish and Mouse Inner Ear Non-sensory Supporting Cells
Source: Front Neurosci. 2019 Oct 18;13:1117. doi: 10.3389/fnins.2019.01117 (PMC6813431; doi:10.3389/fnins.2019.01117)
Supplement: Supplementary file 7 [file Data_Sheet_3.pdf]

S3 File: ShinyGO Analyses of Enriched Biological Processes in Supporting Cell Populations

Common Downregulated Genes in nsSCs, PCs and DCs (n = 306)

Enrichment FDR	Genes in list	Functional Category
1.54E-04	45	GO:0034613 cellular protein localization
1.54E-04	45	GO:0070727 cellular macromolecule localization
4.82E-03	46	GO:0045184 establishment of protein localization
4.82E-03	9	GO:0048489 synaptic vesicle transport
4.82E-03	14	GO:0060271 cilium morphogenesis
4.82E-03	9	GO:0097480 establishment of synaptic vesicle localization
5.23E-03	35	GO:0030030 cell projection organization
5.42E-03	28	GO:0006886 intracellular protein transport
5.42E-03	9	GO:0097479 synaptic vesicle localization
5.42E-03	29	GO:1902580 single-organism cellular localization
7.40E-03	9	GO:0007605 sensory perception of sound
7.40E-03	32	GO:0016192 vesicle-mediated transport
7.40E-03	25	GO:0048858 cell projection morphogenesis
9.31E-03	31	GO:0007010 cytoskeleton organization
9.31E-03	10	GO:0051650 establishment of vesicle localization
9.63E-03	25	GO:0032990 cell part morphogenesis
9.63E-03	34	GO:0046907 intracellular transport
9.63E-03	41	GO:0051649 establishment of localization in cell
1.25E-02	9	GO:0050954 sensory perception of mechanical stimulus
1.41E-02	9	GO:0044782 cilium organization
1.41E-02	10	GO:0051648 vesicle localization
1.41E-02	7	GO:0099003 vesicle-mediated transport in synapse
1.41E-02	38	GO:1902589 single-organism organelle organization
1.89E-02	20	GO:0007017 microtubule-based process
2.16E-02	39	GO:0015031 protein transport
2.16E-02	31	GO:0032989 cellular component morphogenesis
2.49E-02	29	GO:0000902 cell morphogenesis
2.49E-02	12	GO:0030031 cell projection assembly
2.83E-02	19	GO:0007610 behavior
2.84E-02	2	GO:0019355 nicotinamide nucleotide biosynthetic process from aspartate

Common Upregulated Genes in nsSCs, PCs and DCs (n = 314)

Enrichment FDR	Genes in list	Functional Category
1.65E-06	35	GO:0007167 enzyme linked receptor protein signaling pathway
3.82E-06	56	GO:0008283 cell proliferation
5.31E-06	26	GO:0007169 transmembrane receptor protein tyrosine kinase signaling pathway
1.99E-05	47	GO:0042127 regulation of cell proliferation
1.17E-04	26	GO:0001944 vasculature development
1.40E-04	25	GO:0001568 blood vessel development
1.40E-04	26	GO:0072358 cardiovascular system development
1.68E-04	48	GO:0009888 tissue development
3.38E-04	18	GO:0050673 epithelial cell proliferation
3.42E-04	44	GO:0007155 cell adhesion
3.42E-04	52	GO:0071310 cellular response to organic substance
3.42E-04	32	GO:0072359 circulatory system development
3.51E-04	44	GO:0022610 biological adhesion
3.51E-04	16	GO:0050678 regulation of epithelial cell proliferation
3.57E-04	10	GO:0001935 endothelial cell proliferation
7.58E-04	9	GO:0001936 regulation of endothelial cell proliferation
9.50E-04	41	GO:0009719 response to endogenous stimulus
9.50E-04	32	GO:0051241 negative regulation of multicellular organismal process
9.54E-04	29	GO:0033993 response to lipid
1.10E-03	22	GO:0030036 actin cytoskeleton organization
1.23E-03	32	GO:0060429 epithelium development
1.34E-03	22	GO:0008285 negative regulation of cell proliferation
1.34E-03	13	GO:0040013 negative regulation of locomotion
1.34E-03	26	GO:0051270 regulation of cellular component movement
1.40E-03	13	GO:0051271 negative regulation of cellular component movement
1.71E-03	25	GO:0040012 regulation of locomotion
1.86E-03	8	GO:0046916 cellular transition metal ion homeostasis
1.90E-03	11	GO:0031960 response to corticosteroid
1.90E-03	22	GO:0048598 embryonic morphogenesis
2.08E-03	33	GO:0016477 cell migration

Common Upregulated Genes in nsSCs and DCs (n = 148)

Enrichment FDR	Genes in list	Functional Category
2.19E-05	36	GO:0007155 cell adhesion
2.19E-05	36	GO:0022610 biological adhesion
2.19E-05	28	GO:0051241 negative regulation of multicellular organismal process
9.38E-05	36	GO:0009888 tissue development
1.29E-04	28	GO:0016477 cell migration
3.21E-04	24	GO:0009887 animal organ morphogenesis
3.48E-04	11	GO:0030198 extracellular matrix organization
3.48E-04	11	GO:0043062 extracellular structure organization
3.48E-04	28	GO:0048870 cell motility
3.48E-04	14	GO:0050673 epithelial cell proliferation
3.48E-04	28	GO:0051674 localization of cell
4.39E-04	30	GO:0040011 locomotion
8.60E-04	12	GO:0031589 cell-substrate adhesion
1.33E-03	31	GO:0006928 movement of cell or subcellular component
1.33E-03	32	GO:2000026 regulation of multicellular organismal development
1.77E-03	12	GO:0001667 ameboidal-type cell migration
1.77E-03	22	GO:0046903 secretion
1.93E-03	20	GO:0032940 secretion by cell
2.14E-03	35	GO:0048468 cell development
2.14E-03	23	GO:0060429 epithelium development
2.74E-03	28	GO:0022008 neurogenesis
3.14E-03	11	GO:0050678 regulation of epithelial cell proliferation
4.09E-03	22	GO:0009790 embryo development
4.09E-03	6	GO:0034446 substrate adhesion-dependent cell spreading
4.09E-03	8	GO:0050679 positive regulation of epithelial cell proliferation
5.09E-03	17	GO:0045596 negative regulation of cell differentiation
5.37E-03	8	GO:0043491 protein kinase B signaling
7.35E-03	15	GO:0061061 muscle structure development
7.38E-03	3	GO:0002576 platelet degranulation
7.38E-03	16	GO:0051046 regulation of secretion

Common Upregulated Genes in nsSCs and PCs (n = 121)

Enrichment FDR	Genes in list	Functional Category
3.52E-05	29	GO:0007155 cell adhesion
3.52E-05	26	GO:0009967 positive regulation of signal transduction
3.52E-05	29	GO:0022610 biological adhesion
3.52E-05	18	GO:0030029 actin filament-based process
3.52E-05	17	GO:0030036 actin cytoskeleton organization
5.15E-05	27	GO:0010647 positive regulation of cell communication
5.15E-05	27	GO:0023056 positive regulation of signaling
6.67E-05	23	GO:0007010 cytoskeleton organization
6.67E-05	5	GO:0031579 membrane raft organization
6.67E-05	20	GO:0061024 membrane organization
7.90E-05	28	GO:0044093 positive regulation of molecular function
1.05E-04	24	GO:0048870 cell motility
1.05E-04	24	GO:0051674 localization of cell
1.15E-04	29	GO:0048584 positive regulation of response to stimulus
1.15E-04	27	GO:1902589 single-organism organelle organization
1.33E-04	22	GO:0098609 cell-cell adhesion
1.53E-04	27	GO:0006928 movement of cell or subcellular component
1.53E-04	24	GO:0043085 positive regulation of catalytic activity
1.53E-04	26	GO:1902531 regulation of intracellular signal transduction
1.61E-04	11	GO:0031589 cell-substrate adhesion
1.77E-04	22	GO:0016477 cell migration
1.77E-04	25	GO:0040011 locomotion
2.20E-04	23	GO:0001932 regulation of protein phosphorylation
2.31E-04	26	GO:0031399 regulation of protein modification process
2.40E-04	26	GO:0019220 regulation of phosphate metabolic process
2.40E-04	26	GO:0051174 regulation of phosphorus metabolic process
2.49E-04	6	GO:0032963 collagen metabolic process
2.49E-04	21	GO:0071495 cellular response to endogenous stimulus
2.54E-04	25	GO:0009719 response to endogenous stimulus
2.54E-04	6	GO:0044259 multicellular organismal macromolecule metabolic process

High-confidence orthologs upregulated in PC and DC, and down in nsSCs (n = 72)

Enrichment FDR	Genes in list	Functional Category
1.08E-03	7	GO:0048754 Branching morphogenesis of an epithelial tube
1.57E-03	4	GO:0001569 Branching involved in blood vessel morphogenesis
1.57E-03	7	GO:0001763 Morphogenesis of a branching structure
1.57E-03	7	GO:0061138 Morphogenesis of a branching epithelium
2.86E-03	8	GO:0060562 Epithelial tube morphogenesis
4.30E-03	10	GO:0048729 Tissue morphogenesis
5.08E-03	9	GO:0002009 Morphogenesis of an epithelium
6.78E-03	12	GO:0009887 Animal organ morphogenesis
8.94E-03	20	GO:0009653 Anatomical structure morphogenesis
1.03E-02	7	GO:0003002 Regionalization
1.32E-02	2	GO:2000096 Positive regulation of Wnt signaling pathway, planar cell polarity pathway
1.65E-02	11	GO:1901135Carbohydrate derivative metabolic process
1.65E-02	23	GO:0048513 Animal organ development
1.65E-02	3	GO:0060071 Wnt signaling pathway, planar cell polarity pathway
1.65E-02	4	GO:0060993 Kidney morphogenesis
1.65E-02	3	GO:0090175 Regulation of establishment of planar polarity
1.91E-02	4	GO:1905330 Regulation of morphogenesis of an epithelium
1.91E-02	5	GO:2000027 Regulation of animal organ morphogenesis
2.10E-02	2	GO:2000052 Positive regulation of non-canonical Wnt signaling pathway
2.10E-02	6	GO:0007265 Ras protein signal transduction
2.10E-02	7	GO:0007389 Pattern specification process
2.10E-02	17	GO:0007399 Nervous system development
2.10E-02	5	GO:0009952 Anterior/posterior pattern specification
2.10E-02	14	GO:0022008 Neurogenesis
2.10E-02	3	GO:0035567 Non-canonical Wnt signaling pathway
2.16E-02	12	GO:0007267 Cell-cell signaling
2.20E-02	4	GO:0030177 Positive regulation of Wnt signaling pathway
2.21E-02	2	GO:2000095 Regulation of Wnt signaling pathway, planar cell polarity pathway
2.21E-02	10	GO:0007417 Central nervous system development
2.43E-02	10	GO:0000902 Cell morphogenesis

Unique upregulated gene orthologs in nsSCs (n = 1,556)

Enrichment FDR	Genes in list	Functional Category
6.55E-25	46	GO:0043043 Peptide biosynthetic process
6.55E-25	46	GO:0006412 Translation
4.73E-24	46	GO:0006518 Peptide metabolic process
1.31E-23	46	GO:0043604 Amide biosynthetic process
1.03E-20	46	GO:0043603 Cellular amide metabolic process
5.46E-20	61	GO:1901566 Organonitrogen compound biosynthetic process
2.27E-17	97	GO:0010467 Gene expression
7.26E-17	115	GO:0034641 Cellular nitrogen compound metabolic process
2.77E-14	87	GO:0034645 Cellular macromolecule biosynthetic process
3.90E-14	99	GO:0044249 Cellular biosynthetic process
4.60E-14	88	GO:0044271 Cellular nitrogen compound biosynthetic process
5.25E-14	101	GO:0009058 Biosynthetic process
6.34E-14	87	GO:0009059 Macromolecule biosynthetic process
9.52E-14	99	GO:1901576 Organic substance biosynthetic process
2.41E-11	47	GO:0009790 Embryo development
2.92E-10	100	GO:0032502 Developmental process
5.48E-10	93	GO:0007275 Multicellular organism development
8.73E-10	29	GO:0043009 Chordate embryonic development
9.09E-10	29	GO:0009792 Embryo development ending in birth or egg hatching
9.48E-10	97	GO:0048856 Anatomical structure development
1.88E-08	80	GO:0048731 System development
2.09E-07	54	GO:0009653 Anatomical structure morphogenesis
2.62E-07	84	GO:0044267 Cellular protein metabolic process
2.76E-07	63	GO:0048513 Animal organ development
1.55E-06	96	GO:0032501 Multicellular organismal process
3.30E-06	12	GO:0048872 Homeostasis of number of cells
3.55E-06	103	GO:1901564 Organonitrogen compound metabolic process
7.41E-06	28	GO:0035295 Tube development
1.01E-05	24	GO:0035239 Tube morphogenesis
1.10E-05	28	GO:0048646 Anatomical structure formation involved in morphogenesis
